# Longitudinal association between parent–child relationship and depression among Chinese adolescents: the role of psychological resilience and school climate

**DOI:** 10.1186/s13034-025-00952-y

**Published:** 2025-08-25

**Authors:** Zhi-Ying Zeng, Wan-Yu Ye, Yu-Zhe He, Wen-Hao Gu, Sheng-Nan Li, Yan-Gang Nie

**Affiliations:** Department of Psychology and Research Center of Adolescent Psychology and Behavior, Guangzhou University. Guangzhou Higher Education Mega Center, Waihuan Road West, 230 Panyu District, Guangzhou, People’s Republic of China

**Keywords:** Adolescence, Parent–child relationship, Depression, Psychological resilience, School climate

## Abstract

**Background:**

The relationship between the parent–child relationship and adolescent depression is much discussed, but still not fully understood. Based on ecological systems theory, this study examined the potential mediating role of psychological resilience and the moderating role of school climate between the association of parent–child relationship and adolescent depression.

**Methods:**

This study employed a three-wave longitudinal design with six months between each time point, involving 549 elementary and middle school students in southeastern China (50.82% males; mean age at Time 1 = 11.43). Measurements included the parent–child relationship (T1), psychological resilience (T2), school climate (T3), depression (T1 and T3), and demographic information.

**Results:**

The moderated mediation model demonstrated that after controlling for baseline adolescent depression (T1), the parent–child relationship (T1) was longitudinally and negatively associated with adolescent depression (T3) through the mediating effect of psychological resilience (T2). Additionally, the analysis revealed that a positive school climate moderated this mediation by mitigating the adverse impact of low psychological resilience on adolescent depression, thus reducing the indirect effect of the parent–child relationship on adolescent depression.

**Conclusions:**

Our findings offer a nuanced understanding of the underlying mechanisms linking parent–child relationship to adolescent depression among Chinese adolescents. Theoretical contributions and practical applications of these findings are further elaborated.

**Supplementary Information:**

The online version contains supplementary material available at 10.1186/s13034-025-00952-y.

## Background

Adolescent depression has become a major concern in the field of global public health. It detrimentally affects academic performance, interpersonal relationships, and physical and mental health, and may also exert long-term effects on their social functioning in adulthood [2, 18]. Within the family environment, the parent–child relationship is considered a crucial factor influencing adolescents' psychological development and mental health [66]. A large body of research has shown that the parent–child relationship is closely associated with adolescents' depression [26, 54]. Positive parent–child interactions, characterized by warmth, support, and trust, can provide emotional security, enhance interpersonal trust, and serve as a protective factor against depression [47, 55]. In contrast, parent–child conflict, emotional neglect, or controlling parenting styles may increase adolescents' vulnerability to psychological distress, especially when they encounter academic or life stressors [39, 54]. Bronfenbrenner's [7] ecological model highlights the family as the most influential and immediate aspect of the ecological environment in human development. A negative family environment can result in maladaptive developmental outcomes [28, 51]. Therefore, exploring the mechanisms linking parent–child relationship with adolescent depression is essential for understanding the development of depression and designing effective intervention programs.

Previous studies have established a direct link between parent–child relationship and adolescent depression [14], yet the mediating and moderating mechanisms underlying this association have yet to be fully explored. Psychological resilience helps adolescents cope with adversity and may mediate the link between parent–child relationships and depression. Positive parent–child interactions enhance emotional support and self-regulation, reducing depression, while conflict or neglect weaken resilience and increase depression risk [8, 44]. Moreover, school climate, as an external factor, can mitigate the impact of family stressors on adolescent mental health [43]. A supportive school environment enhances resilience and reduces the risk of depression, whereas a negative school climate may weaken the protective effects of resilience. This study adopts a moderated mediation model to examine psychological resilience as a mediator between parent–child relationship and adolescent depression, and to explore how school climate moderates this process, offering deeper insight into the interplay between family and school contexts in shaping adolescent mental health.

## The influence of the parent–child relationship on adolescent depression

A positive parent–child relationship serves as an important protective factor for adolescent mental health, significantly reducing levels of depression. Warm and highly supportive parent–child interactions promote adolescents' emotional regulation abilities and positive self-cognition, thereby lowering the risk of depression [49]. In contrast, parent–child conflict or emotional estrangement markedly increases the risk of depression by fostering emotional deprivation, cognitive biases, and feelings of helplessness [41, 46]. Research has demonstrated a bidirectional relationship between parent–child conflict and adolescents' depression, particularly during early adolescence, when family dynamics are highly influential [31]. Moreover, the quality of the parent–child relationship can moderate the effects of environmental stressors, such as family chaos, on adolescents' mental health by enhancing emotional security [34]. Studies have further indicated that a parent–child relationship characterized by high support and low conflict is associated with lower levels of antisocial behavior and depression among adolescents [45]. Importantly, the impact of the parent–child relationship extends beyond adolescence, influencing mental health outcomes into adulthood. For instance, strong family and peer support during adolescence can buffer the effects of early life stress on later depression [52]. In summary, strengthening support and reducing conflict within the parent–child relationship represents a crucial strategy for preventing and alleviating adolescent depression [47, 60].

## The mediation effect of psychological resilience

Adolescence is a critical developmental period during which individuals are particularly vulnerable to environmental stressors, making the cultivation of psychological resilience essential for mental well-being [42]. Defined as an individual’s ability to adapt and recover effectively in the face of adversity [27], psychological resilience is influenced by both individual traits and the family environment. Positive parent–child relationship fosters the development of psychological resilience by providing emotional support, problem-solving guidance, and role modeling [67]. Research indicates that high-quality communication and emotional connections between parents and adolescents enhance adolescents’ sense of security and confidence in coping, thereby improving their psychological adaptability when facing stressful situations [61]. Moreover, psychological resilience serves as a protective factor that buffers the adverse effects of negative life events such as family conflict and peer bullying on adolescents [3, 25]. Importantly, resilience also mediates the relationship between family adaptability, school-related stressors, and adolescent depression, with evidence indicating that supportive interpersonal relationships bolster resilience and subsequently reduce depression [21]. Collectively, these findings highlight psychological resilience as a crucial resource enabling adolescents to navigate challenges across familial and educational domains and thereby sustain their mental health. Consequently, interventions aimed at strengthening resilience hold promise as effective strategies for mitigating depression risk during adolescence.

## The moderation effect of school climate

School is a vital developmental context that adolescents engage with daily, playing a pivotal role in shaping mental health outcomes through its interaction with internal psychological factors [7]. Defined as the supportive, caring atmosphere and sense of belonging that students perceive within their school environment [40], school climate not only directly influences adolescents' mental health but also moderates the relationship between internal resources and psychological outcomes. Specifically, a positive school climate can enhance the protective effect of psychological resilience on depression [24, 32, 33].

Research indicates that while psychological resilience generally serves as a buffer against depression, its effectiveness can vary depending on the surrounding environment [48]. Supportive school climate can provide interpersonal and emotional resources that individuals with resilience can utilize to mitigate depression [15, 37]. Furthermore, studies have demonstrated that positive school environments are associated with lower levels of internalizing and externalizing problems through their promotive effect on resilience among both children and adolescents [11, 43]. In contrast, an adverse school climate may diminish the protective function of psychological resilience, thereby reducing its effectiveness in mitigating depression [24]. In negative school climates characterized by insufficient teacher support and elevated peer conflict, adolescents may be deprived of critical emotional resources, thereby undermining their capacity to develop and sustain psychological resilience [13, 59].

Based on these findings, we propose that a positive school climate may strengthen the protective effect of psychological resilience against depression, whereas a negative school climate may weaken this protective effect.

## The current study

**Fig. 1:**
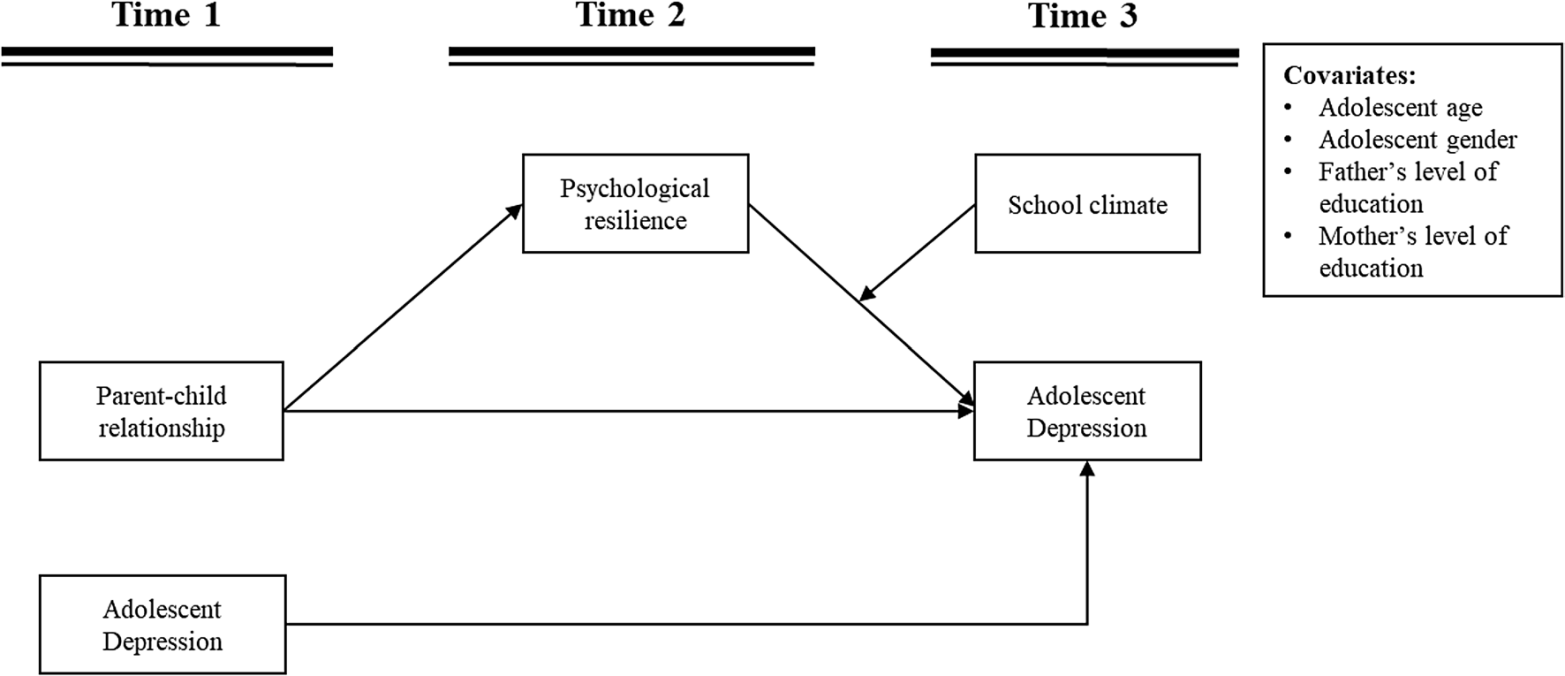
Conceptual moderated mediation model

Based on ecological systems theory, this study adopts a comprehensive approach that integrates family, individual, and school perspectives to examine the mechanisms influencing adolescent depression. Specifically, the research investigates the longitudinal relationships between parent–child dynamics and adolescent depression within a Chinese sample, while also exploring the roles of psychological resilience and school climate. Using data collected at three time points, separated by six-month intervals, the study tests a longitudinal model incorporating the processes illustrated in Fig. 1. Parent–child relationship (T1), psychological resilience (T2), school climate (T3), and depression (T1/T3) were assessed through self-reported questionnaires. Additionally, control variables, including adolescent age, gender, and parental education levels, were included in the analysis.

We hypothesized that: parent–child relationship is associated negatively with adolescent depression (Hypothesis 1); psychological resilience will mediate the association between parent–child relationship and adolescent depression (Hypothesis 2); school climate would moderate the association between psychological resilience and adolescent depression (Hypothesis 3a), and school climate would moderate the mediation effect of psychological resilience (Hypothesis 3b).

## Method

### Participants and procedure

A total of 549 students (279 boys, 270 girls) were enrolled via convenience sampling from two elementary schools and three middle schools in Guangzhou, China. At the first assessment (T1; October 2018), participants ranged in age from 8 to 15 years (*M*_age_ = 11.43, *SD* = 1.55). Fathers’ educational attainment was distributed as follows: 24.7% had a primary degree, 25.7% had a middle school degree, 33.6% had earned a college degree or equivalent, and 16.0% had earned a graduate degree; mothers’ education comprised 33.6% had a primary degree, 25.2% had a middle school degree, 27.3% had earned a college degree or equivalent, and 13.9% had earned a graduate degree. Six months later (T2; April 2019), 539 adolescents (275 boys, 264 girls; 98.36% retention) were reassessed using identical procedures. At the third wave (T3; October 2019), 529 participants (265 boys, 264 girls) remained, reflecting an overall attrition of 3.64% due solely to absences on testing days. All students continued attending their originally sampled schools throughout the study.

Ethical clearance was obtained from the Institutional Review Board of the lead author’s institution (No. GZHU2019007). Before data collection, participants provided verbal assent, and their guardians gave written consent; all participants were free to withdraw at any time without penalty. Trained assistants followed a standardized protocol to administer assessments in classrooms during regular hours at each predefined time point. No incentives were offered.

## Measures

### Parent–child relationship

Parent–child relationship was assessed at T1 using the Closeness to Parents Scale (CPS; [9]) to evaluate adolescents perceived relational quality with both fathers and mothers. The scale comprises two parallel subscales (9 items each) measuring *father-child closeness* and *mother–child closeness*, respectively. Participants rated statements such as “*How openly do you talk with your [mother/father]?*” on a 5-point Likert scale (1 = *Not at all* to 5 = *Strongly agree*). Subscale scores were calculated by averaging responses, with higher scores reflecting greater perceived relational quality. The Chinese CPS has established validity and reliability in adolescent samples [62]. In the current sample, internal consistency was excellent: Cronbach’s *α* = 0.890 (total), 0.848 (father-child relationship), and 0.831 (mother–child relationship).

### Psychological resilience

Psychological resilience was assessed at T2 using the 13-item Resilience Scale-13 (RS-13; [36]), a validated short-form adaptation of the original Resilience Scale-14 (RS-14, [53]). Participants rated statements (e.g., “I usually take things in stride.”) on a 7-point Likert scale (1 = *Strongly disagree* to 7 = *Strongly agree*). A composite resilience score was derived by averaging all items, with higher scores indicating greater psychological resilience. The Chinese version of the scale has demonstrated good internal consistency in adolescents [64]. In the current sample, the scale exhibited excellent internal consistency (Cronbach’s *α* = 0.933).

### School climate

School climate was measured at T3 using the School Climate Questionnaire [5, 6]. This 28-item instrument evaluates seven core dimensions of the school environment: teacher-student relations (5 items), student–student relations (4 items), school-wide student engagement (5 items), clarity of behavioral expectations (4 items), fairness of institutional rules (4 items), perceived safety (3 items), and school-wide bullying prevalence (3 items). Sample items included “School is safe”, rated on a 5-point Likert scale (1 = *Very inconsistent* to 5 = *Very consistent*). A composite score was derived by averaging responses across all items, with higher scores indicating stronger perceptions of a positive school climate. The Chinese version of the scale has demonstrated good internal consistency in adolescents [23, 58]. The Cronbach’s *α* in this study was 0.934.

### Depression

Depression was assessed at baseline (T1) and follow-up (T3) using the 10-item Chinese adaptation of the Center for Epidemiologic Studies Depression Scale (CES-D; [4]). Respondents rated symptom frequency over the preceding week on a 4-point Likert scale (1 = *Rarely or none of the time* to 4 = *Most or all of the time*), with higher scores reflecting greater symptom severity. Reverse-coded items (e.g., “I felt hopeful about the future”) were transformed before calculating mean scores. A sample item included “I felt lonely”. The Chinese version of the scale demonstrated good internal consistency [50, 56], with Cronbach’s *α* = 0.850 at Time 1 and Cronbach’s *α* = 0.716 at Time 3 in the present study.

### Demographic covariates

Considering their established association with adolescent depression [10, 35], adolescent gender, adolescent age, and both mother’s and father’s education were included as covariates in all analyses. Categorical variables were dummy coded before analysis. Gender was coded as a binary variable (0 = male [reference], 1 = female), and both mother’s and father’s education levels were dummy coded using “primary school and below” as the reference category, with separate indicators for middle school, undergraduate, and graduate degrees.

## Analytic strategies

A total of 5.82% of observations were missing across the three assessment waves and were handled via full-information maximum likelihood estimation under the assumption that data were missing at random [1]. We began by evaluating sample attrition, computing descriptive statistics, and examining bivariate correlations for all study variables in IBM SPSS Statistics 26.0. Next, we assessed the potential impact of common-method variance given our reliance on self-report instruments. Thereafter, we specified a series of structural equation models in Mplus Version 8.3 [30]. To evaluate Hypotheses 1 and 2, we estimated a mediation model in which T1 parent–child relationship served as the predictor, T2 psychological resilience as the mediator, and T3 adolescent depression as the outcome. Subsequently, to test Hypotheses 3a and 3b, we expanded the model to include T3 school climate as a moderator of the relationship between psychological resilience and adolescent depression. All continuous variables were mean‐centered before forming interaction terms. Whenever a significant interaction emerged, we conducted simple-slope analyses at one standard deviation above and below the moderator’s mean and examined the indirect effect of T1 parent–child relationship on T3 adolescent depression via T2 psychological resilience at these conditional levels of school climate.

The adequacy of the model was assessed using the following indices: the Comparative Fit Index (CFI; acceptable values > 0.90), the Root Mean Square Error of Approximation (RMSEA; acceptable values < 0.08), and the Standardized Root Mean Square Residual (SRMR; acceptable values < 0.08). Given the advantages of bootstrapping over traditional methods for testing mediation models [38], we employed a bootstrapping technique with 5,000 resamples and 95% confidence intervals (CI) to evaluate both indirect and direct effects. A (moderated) mediation effect was deemed statistically significant when the 95% CI did not include zero.

## Results

### Attrition analysis

We conducted attrition analyses to compare participants with complete data across all waves (completer group) and those with missing data at T2 and/or T3 (missing group). Independent-samples t-tests indicated no significant differences between the two groups on Time 1 father–child relationship (*t* (547) = 1.76, *p* = 0.078), Time 1 mother–child relationship (*t* (547) = 1.79, *p* = 0.074), or Time 1 adolescent depression (*t* (547) = –1.30, *p* = 0.193). Chi-square tests likewise revealed equivalence in gender distribution (*χ*^2^ (1) = 0.995,* p* = 0.318), fathers’ education level (*χ*^2^ (3) = 2.84, *p* = 0.417), and mothers’ education level (*χ*^2^ (3) = 7.25, *p* = 0.123). The only significant difference emerged for age, with the missing group (*M* = 11.97, *SD* = 1.49) being older than the completer group (*M* = 11.39, *SD* = 1.55; *t* (547) = –2.04, *p* < 0.05). Little’s test for Missing Completely at Random (MCAR) yielded *χ*^2^ (4) = 8.29, *p* = 0.082, indicating that the pattern of missing data can be treated as random and is unlikely to bias parameter estimates.

## Common method bias

Because all measures were self-reported by adolescents, we examined the potential for common method bias using the classic Harman single-factor test. An unrotated exploratory factor analysis of all items yielded a first factor that explained only 21.97% of the total variance, well below the 50% threshold. These findings indicate that common method bias does not pose a serious threat to our results.

## Descriptive and correlational

Descriptive statistics and bivariate correlations for key variables are summarized in Table 1. Specifically, the T1 parent–child relationship (both paternal and maternal) was negatively related to T1/ T3 adolescent depression and positively related to T2 psychological resilience and T3 school climate, respectively. Besides, T2 psychological resilience was negatively related to T1/T3 adolescent depression and positively related to T3 school climate, respectively. Moreover, T3 school climate was negatively related to T1/T3 adolescent depression, respectively.

**Table 1:** The means, standard deviations, correlations among the variables

	*M*	*SD*	1	2	3	4	5	6	7	8	9
Covariates											
1. Adolescent age at T1	11.43	1.55									
2. Adolescent gender	49.18^a^	–	− 0.07								
3. Father’s level of education	–	–	− 0.62^***^	0.04							
4. Mother’s level of education	–	–	− 0.61^***^	0.04	0.99^***^						
Key variables											
5. T1 Father-child relationship	3.55	0.84	− 0.23^***^	0.04	0.09^*^	0.08					
6. T1 Mother–child relationship	3.86	0.75	− 0.26^***^	0.08	0.13^**^	0.14^**^	0.59^***^				
7. T2 Psychological resilience	4.95	1.08	− 0.12^**^	− 0.01	0.13^**^	0.13^**^	0.16^***^	0.20^***^			
8. T3 School climate	3.12	0.36	− 0.25^***^	0.04	0.22^***^	0.21^***^	0.30^***^	0.31^***^	0.32^***^		
9. T1 Depression	0.93	0.50	0.12^**^	− 0.02	− 0.01	0.01	− 0.23^***^	− 0.19^***^	− 0.22^***^	− 0.21^***^	
10. T3 Depression	0.85	0.48	0.28^***^	0.06	− 0.22^***^	− 0.21^***^	− 0.30^***^	− 0.30^***^	− 0.33^***^	− 0.40^***^	0.35^***^

*Note* Sample size ranged from 529 to 549 due to missing data. ^*^ *p* < 0.05, ^**^ *p* < 0.01, ^***^p < 0.001. Adolescent gender and parental education were included as dummy-coded covariates (see Method section). ^a^ The percentage of female adolescents; T1 = Time 1, T2 = Time 2, T3 = Time 3

## Mediating effects of psychological resilience

**Fig. 2:**
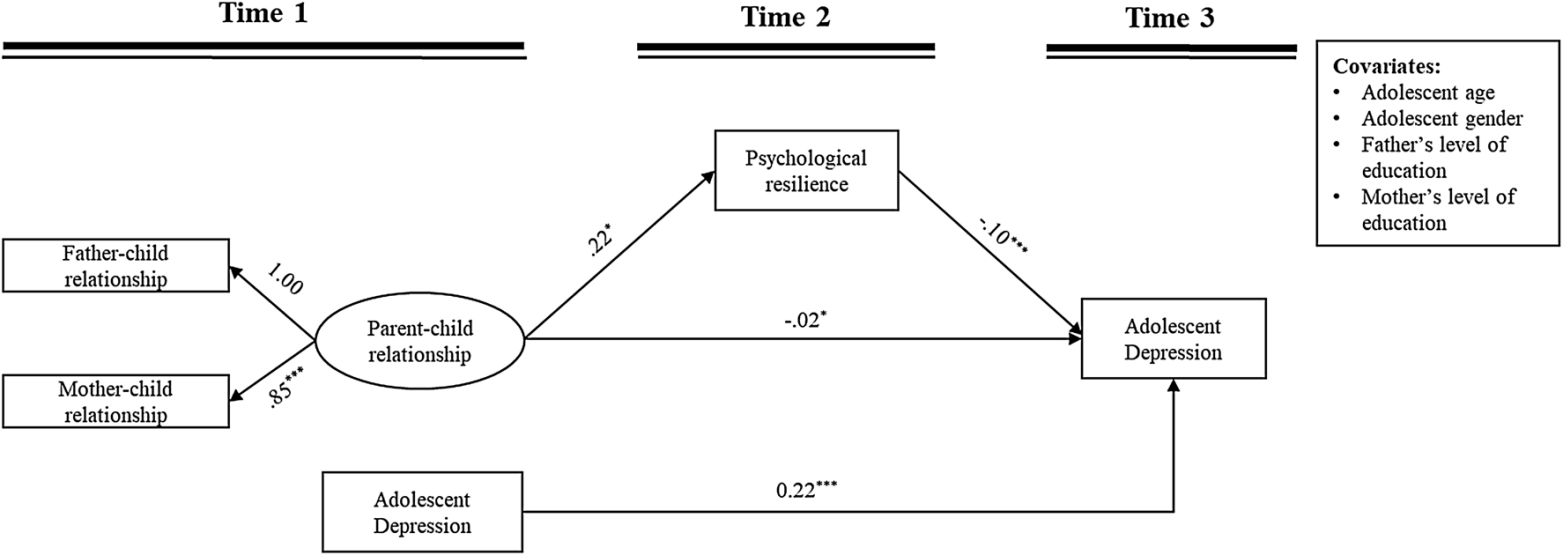
The mediating effect of T2 psychological resilience in the relation between T1 parent–child relationship and T3 adolescent depression. *Note* Unstandardized coefficients are reported; ^*^p < 0.05, ^***^p < 0.001

The mediation model demonstrated a good fit to the data: χ^2^ = 26.09, *df* = 12, *p* < 0.05; RMSEA = 0.056 with a 90% CI, [0.026, 0.085]; CFI = 0.970 and SRMR = 0.035. As shown in Fig. 2 and Table 2, T1 parent–child relationship was positively associated with T2 psychological resilience, which was in turn negatively associated with T3 depression. The indirect effect of T1 parent–child relationship on T3 depression via T2 psychological resilience was statistically significant.

**Table 2:** *S*ummary of the direct and indirect effects

Direct and indirect effects	Bias-corrected bootstrapped estimates for the effects			
	*B*	*SE*	95% CI	β
Direct pathway				
T1 Parent–child relationship → T3 Adolescent depression	− **0.21**	**0.05**	**[**− **0.316, **− **0.113]**	− **0.31**
Indirect pathways				
T1 Parent–child relationship → T2 Psychological resilience → T3 Adolescent depression	− **0.02**	**0.01**	**[**− **0.050, **− **0.004]**	− **0.03**

*Note* T1 = Time 1, T2 = Time 2, T3 = Time 3; The significant results are in bold. *B* = unstandardized coefficient; *SE* = standard error; CI = confidence interval for the standardized coefficient; β = standardized coefficient

## Moderating effects of school climate

**Fig. 3:**
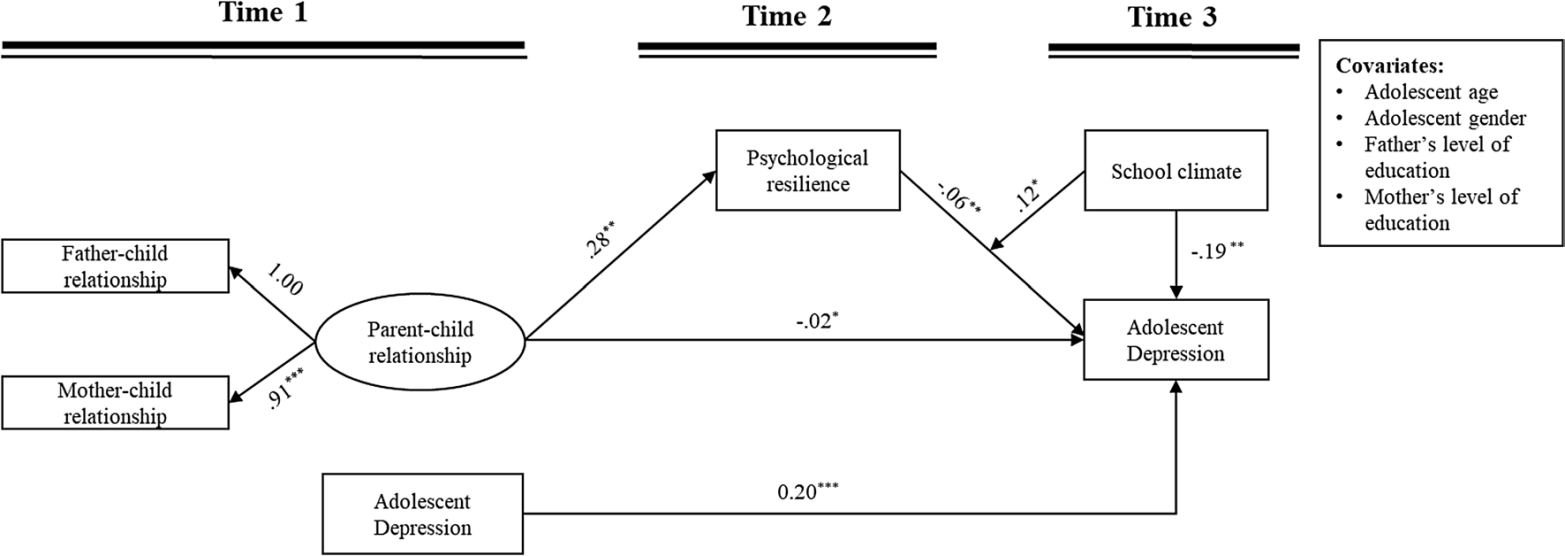
Moderated mediation model. *Note* Unstandardized coefficients are reported; ^*^p < 0.05, ^**^p < 0.01, ^***^p < 0.001

Based on the testing for the mediation model, we continued to examine whether T3 school climate would moderate the relation between T2 psychological resilience and T3 adolescent depression, as well as the mediating effect of T2 psychological resilience. The moderated mediation model implied a good fit to the data: χ^2^ = 55.53, *df* = 18, *p* < 0.001; RMSEA = 0.077 with a 90% CI = [0.054, 0.100]; CFI = 0.922 and SRMR = 0.042. The results are displayed in Table 3 and Fig. 3, which suggests that T3 school climate moderated the relation between T2 psychological resilience and T3 adolescent depression.

**Table 3:** Summary of the moderated mediation model

	T2 Psychological resilience (*R*^2^ = 0.08)				T3 Adolescent depression (*R*^2^ = 0.32)			
	*B*	*SE*	β	*p*	*B*	*SE*	β	*p*
Covariates								
Adolescent age at T1					0.01	0.02	0.04	0.480
Adolescent gender					0.03	0.04	0.03	0.529
Father’s level of education					0.00	0.03	0.01	0.900
Mother’s level of education					0.00	0.04	0.01	0.926
Key variables								
T1 Parent–child relationship	**0.28**	**0.10**	**0.18**	** < 0.01**	− **0.20**	**0.05**	− **0.29**	** < 0.001**
T2 Psychological resilience					− **0.06**	**0.02**	− **0.14**	** < 0.01**
T3 School climate					− **0.19**	**0.07**	− **0.15**	** < 0.01**
T2 Psychological resilience × T3 School climate					**0.12**	**0.05**	**0.12**	** < 0.05**
T1 Depression					**0.20**	**0.05**	**0.22**	** < 0.001**

*Note* T1 = Time 1, T2 = Time 2, T3 = Time 3; The significant results are in bold

**Fig. 4:**
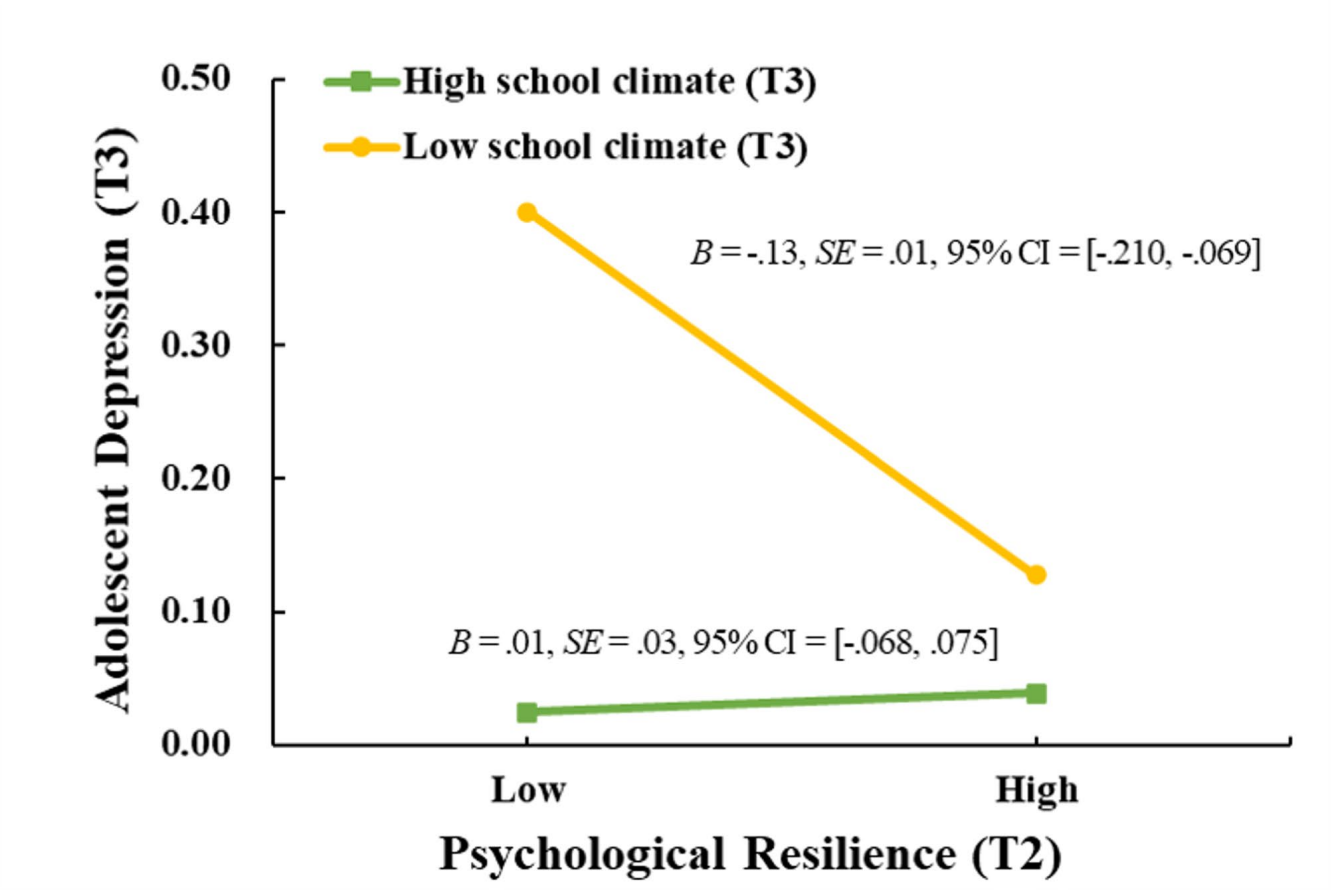
The relation between T2 psychological resilience and T3 adolescent depression by T3 school climate. ***Note:*** T2 = Time 2, T3 = Time 3

As illustrated in Fig. 4, simple slope analysis showed that the negative association between T2 psychological resilience and T3 adolescent depression was stronger under low school climate than under high school climate. As shown in Table 4, the indirect effect of T1 parent–child relationship on T3 depression via T2 psychological resilience was significant only under low school climate, but not under high school climate. To provide a more detailed understanding of the interaction effect, we conducted a Johnson–Neyman analysis, the results of which are presented in Appendix A1.

**Table 4:** Conditional indirect effects of T1 parent–child relationship on T3 adolescent depression via T2 psychological resilience by levels of T3 school climate

Levels of T3 school climate	*B*	*SE*	95% CI
Low (*M−SD*)	− **0.05**	**0.03**	**[**− **0.115, **− **0.012]**
Med (*M*)	− **0.02**	**0.01**	**[**− **0.042, **− **0.004]**
High (*M* + *SD*)	**0.02**	**0.02**	**[**− **0.010, 0.059]**
Diff (High−Low)	**0.06**	**0.04**	**[0.009, 0.168]**

*Note* T1 = Time 1, T2 = Time 2, T3 = Time 3. The significant results are in bold

## Discussion

From the perspective of ecological systems theory [7], this study explored the longitudinal relationship between parent–child relationship and adolescent depression among Chinese adolescents, while also examining the mediating and moderating mechanisms involved. By considering multiple developmental contexts, such as family and school, this research provides a more comprehensive understanding of adolescent depression compared to studies focused on a single context. The key findings showed that psychological resilience played a mediating role in the connection between parent–child relationship and adolescent depression, particularly in the context of a negative school climate. Furthermore, this study employed a longitudinal design to assess the core variables, thereby addressing the limitations of prior research that predominantly utilized cross-sectional methodologies. This approach enhances the potential for causal inference and mitigates common method bias.

## Parent–child relationship and adolescent depression

The results of this study provide empirical support for Hypothesis 1, which posits a negative correlation between parent–child relationship and adolescent depression. Specifically, the study found that positive parent–child relationship is significantly associated with lower levels of adolescent depression. This finding aligns with previous research, indicating that warm and supportive parent–child relationship are crucial protective factors for adolescent mental health [16]. A positive parent–child relationship provides emotional support to adolescents, helping to mitigate the effects of stressors and reduce the risk of depression [63]. Furthermore, the quality of the parent–child relationship also influences adolescents' attention patterns during negative interactions, with low-quality relationships potentially exacerbating depression [17, 54]. The findings of this study further highlight the importance of fostering positive family interactions in the prevention and intervention of adolescent depression.

## The mediating role of psychological resilience

Consistent with Hypothesis 2, this study demonstrated that psychological resilience significantly mediated the relationship between the parent–child relationship and adolescent depression. Adolescents who reported more positive parent–child relationship exhibited higher levels of psychological resilience, which in turn predicted lower levels of depression. This finding aligns with prior research suggesting that psychological resilience serves as a critical protective factor, buffering individuals against emotional distress [27]. Prior research has similarly shown that resilience mitigates the impact of adverse experiences on mental health [29, 65]. Supportive parent–child interactions, including open communication and emotional warmth, have been linked to higher levels of adolescent resilience, which in turn reduce depression [21]. These findings highlight that fostering psychological resilience is a crucial pathway through which supportive parent–child relationship protect against adolescent depression.

## The moderating role of school climate

Consistent with Hypothesis 3a, this study found that school climate moderated the relationship between psychological resilience and adolescent depression. Specifically, the negative association between psychological resilience and depression was stronger under a negative school climate and weaker under a positive one. This suggests that a supportive school environment can enhance the protective effect of resilience against depression, aligning with prior findings emphasizing the critical role of school climate in adolescent mental health [32]. Previous studies have demonstrated that a positive school climate not only directly reduces depression but also promotes psychological resilience, further mitigating depression risk [43]. These findings provide further evidence that the influence of individual psychological factors on adolescent depression may vary depending on environmental contexts such as school climate.

This study supports Hypothesis 3b, showing that school climate moderates the mediating effect of psychological resilience on the relationship between parent–child relationship and adolescent depression. Research shows that negative school climates, characterized by inadequate teacher support, poor peer interactions, and unsafe conditions, can heighten adolescents' risk of depression [59]. In such adverse environments, adolescents may rely more heavily on internal coping resources such as psychological resilience to navigate stressors, including those stemming from family dysfunction [57]. Conversely, positive school environments with supportive relationships and clear structure can offer external emotional and interpersonal resources that help adolescents manage stress, thereby reducing their reliance on internal resilience mechanisms [22]. Additionally, a previous study indicated that supportive school climate can actively promote the development of individual resilience, which in turn protects adolescents from the psychological impact of family-related stressors [11]. ​These results reflect the interdependent roles of family, individual, and school contexts in adolescent mental health, consistent with an ecological systems perspective.

## Theoretical and practical implications

The findings of this study, grounded in ecological systems theory, highlight the cross-level interactions between family, individual, and school contexts in shaping adolescent mental health. The results emphasize the crucial role of parent–child relationship in mitigating adolescent depression, suggesting that interventions should focus on strengthening family bonds, improving communication, and reducing conflicts. The development of psychological resilience serves as a significant mediator in mitigating depression, particularly among adolescents facing familial challenges. Resilience-focused interventions, such as enhancing emotional regulation and coping skills, may help lower depression risks. Furthermore, the moderating role of school climate underscores the importance of creating positive, supportive school environments. Interventions to improve school climate, such as peer support programs and teacher mental health training, can strengthen adolescents' resilience and further reduce their risk of depression. Overall, this study underscores the need for integrated interventions that address both family dynamics and school environments to promote adolescent well-being.

## Limitations and future research directions

Although this study provides valuable insights into the potential factors influencing adolescent depression in China, several limitations should be noted. First, the sample was primarily composed of adolescents from metropolitan areas, excluding rural and small-town populations, as well as special groups such as left-behind children. The representativeness of the sample needs further examination. Second, while a three-wave longitudinal design was used, the results are correlational and do not imply causal relationships. Additionally, parent–child relationship, psychological resilience, and school climate were measured at only one time point, without controlling for baseline levels of resilience and school climate. Future research should carefully consider the temporal nature of these effects and the potential advantages of a cross-lagged design. Third, the indirect effect of psychological resilience was statistically significant but modest in size. Mediation analyses typically produce small effects due to their complex structure, limited power, and strict control needs [12, 19]. Considering the complexity of adolescent depression, future research should incorporate additional mediators within a biopsychosocial framework. Finally, data were collected solely from adolescent self-reports. Although adolescents are best positioned to express their views on parent–child relationship and school climate [20], future studies should employ multiple sources of information (e.g., parent, peer, and teacher reports) to rigorously test the study hypotheses.

## Conclusions

This three-wave longitudinal study revealed that the T1 parent–child relationship predicts lower T3 adolescent depression indirectly through T2 psychological resilience and that this protective pathway is moderated by the T3 school climate, with a positive school climate amplifying resilience’s buffering effect against depression while a negative school climate diminishes it.

## Supplementary Information

Additional file1 (DOCX 70 kb)

## Data Availability

The datasets used and/or analyzed during the current study are available from the corresponding author on reasonable request.
