# Supplementary material for: Longitudinal association between parent–child relationship and depression among Chinese adolescents: the role of psychological resilience and school climate
Source: Child Adolesc Psychiatry Ment Health. 2025 Aug 25;19:97. doi: 10.1186/s13034-025-00952-y (PMC12376727; doi:10.1186/s13034-025-00952-y)
Supplement: Supplementary file 1 — Additional file1 (DOCX 70 kb) [file 13034_2025_952_MOESM1_ESM.docx]

**Longitudinal association between** **parent-child relationship and depression among Chinese adolescents: The role of psychological resilience and school climate**

**Online supplementary files**

**Appendix A1. Johnson–Neyman Analysis**

We applied the Johnson–Neyman (J-N) technique to further examine the moderating role of school climate in the association between psychological resilience and adolescent depression (Hayes, 2018; Montoya, 2018). This method identifies the specific values of the moderator (school climate) at which the effect of the predictor (resilience) on the outcome (depression) becomes statistically significant, thereby clarifying the nature of the interaction. As shown in Figure S1, the effect of resilience on depression was significantly negative when school climate scores were below 3.41, indicating a stronger protective role of resilience in less favorable school climates. At higher levels of school climate, this moderating effect was nonsignificant. These findings are consistent with the results of the pick-a-point method, while offering a more fine-grained depiction of the interaction between psychological resilience, school climate, and adolescent depression.


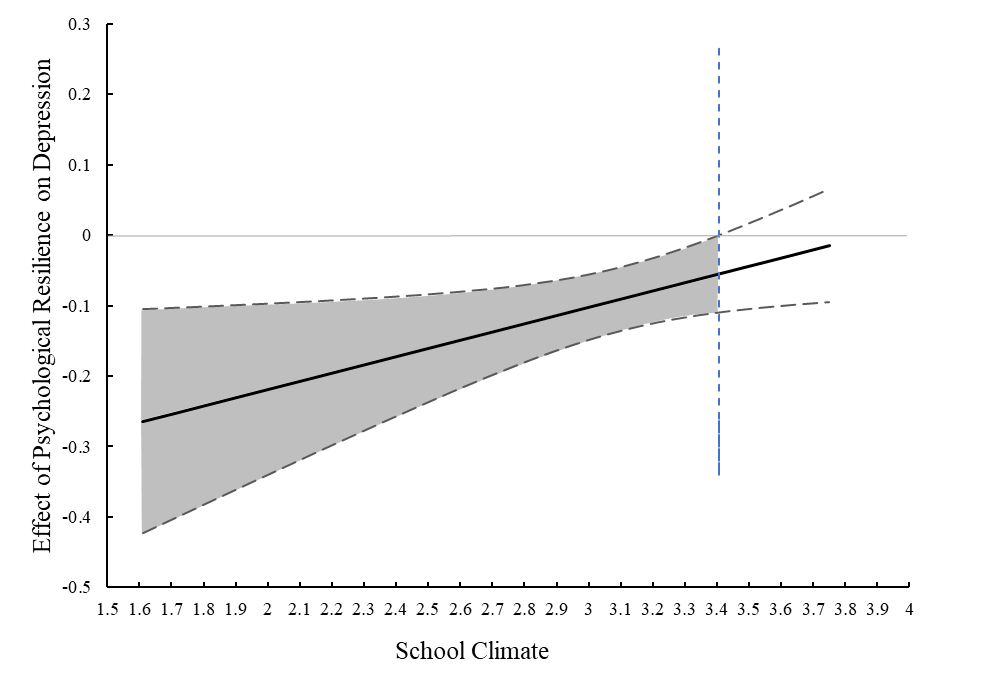


Figure S1. Johnson–Neyman Plot for the Moderating Effect of School Climate on the Association Between Resilience and Depression

***Note:*** The solid black line represents the variation in the effect of resilience on depression across levels of school climate, and the gray dashed lines indicate the 95% confidence intervals. The vertical blue line marks the Johnson–Neyman significance threshold at a school climate value of 3.41. The shaded area to the left of this threshold represents the region where the moderating effect of school climate renders the impact of resilience on depression statistically significant (*p* < .05). Analyses controlled for adolescents’ gender, age, and parental education level.

**Appendix A2. Measures Used in the Study**

**(1) Parent-child relationship**

| **Instructions:**  Please read each question carefully and choose the option that best reflects your experience. Evaluate the statements about your relationship with your parents based on your actual situation, and answer truthfully. | **Not at all** | **Somewhat disagree** | **Neutral** | **Somewhat agree** | **Strongly agree** |
| --- | --- | --- | --- | --- | --- |
| 1. How openly do you talk with your [mother/father]? | 1 | 2 | 3 | 4 | 5 |
| 2. If you needed money, how comfortable would you be asking your [mother/father] for it? | 1 | 2 | 3 | 4 | 5 |
| 3. How comfortable do you feel admitting doubts and fears to your [mother/father]? | 1 | 2 | 3 | 4 | 5 |
| 4. How interested is your [mother/father] in talking to you when you want to talk? | 1 | 2 | 3 | 4 | 5 |
| 5. How often does your [mother/father] express affection or liking for you? | 1 | 2 | 3 | 4 | 5 |
| 6. How well does your [mother/father] know what you are really like? | 1 | 2 | 3 | 4 | 5 |
| 7. How close do you feel to your [mother/father]? | 1 | 2 | 3 | 4 | 5 |
| 8. How confident are you that your [mother/father] would help you if you had a problem? | 1 | 2 | 3 | 4 | 5 |
| 9. How interested is your [mother/father] in the things you do? | 1 | 2 | 3 | 4 | 5 |

**(2) Psychological resilience**

| **Instructions:**  Please read each of the following statements carefully. For each statement, indicate the extent to which you agree or disagree by selecting the number that best represents your feelings. Use the 7-point scale provided, where 1 means 'Strongly Disagree' and 7 means 'Strongly Agree'. There are no right or wrong answers; please respond honestly based on your personal experiences. | **Strongly disagree** | **Disagree** | **Somewhat disagree** | **Neutral** | **Somewhat agree** | **Agree** | **Strongly agree** |
| --- | --- | --- | --- | --- | --- | --- | --- |
| 1. I usually manage one way or another. | 1 | 2 | 3 | 4 | 5 | 6 | 7 |
| 2. I feel proud that I have accomplished things in life. | 1 | 2 | 3 | 4 | 5 | 6 | 7 |
| 3. I usually take things in stride. | 1 | 2 | 3 | 4 | 5 | 6 | 7 |
| 4. I am friends with myself. | 1 | 2 | 3 | 4 | 5 | 6 | 7 |
| 5. I feel that I can handle many things at a time. | 1 | 2 | 3 | 4 | 5 | 6 | 7 |
| 6. I am determined. | 1 | 2 | 3 | 4 | 5 | 6 | 7 |
| 7. I have self-discipline. | 1 | 2 | 3 | 4 | 5 | 6 | 7 |
| 8. I keep interested in things. | 1 | 2 | 3 | 4 | 5 | 6 | 7 |
| 9. I can usually find something to laugh about. | 1 | 2 | 3 | 4 | 5 | 6 | 7 |
| 10. My belief in myself gets me through hard times | 1 | 2 | 3 | 4 | 5 | 6 | 7 |
| 11. In an emergency, I'm someone people can generally rely on. | 1 | 2 | 3 | 4 | 5 | 6 | 7 |
| 12. My life has meaning. | 1 | 2 | 3 | 4 | 5 | 6 | 7 |
| 13. When I'm in a difficult situation, I can usually find my way out of it. | 1 | 2 | 3 | 4 | 5 | 6 | 7 |

**(3) School Climate Scale**

| **Instructions:**  Please read the following description carefully. Choose the appropriate number according to your first feeling. There is no right or wrong answer. Please do not miss any questions, even if you are not sure. | **Strongly disagree** | **Disagree** | **Agree** | **Strongly agree** |
| --- | --- | --- | --- | --- |
| 1. Most students in the class are attentive. | 1 | 2 | 3 | 4 |
| 2. School rules are fair. | 1 | 2 | 3 | 4 |
| 3. School is safe. | 1 | 2 | 3 | 4 |
| 4. Rules are made clear to students. | 1 | 2 | 3 | 4 |
| 5. Most students try their best to be the best (in study, activity, etc.). | 1 | 2 | 3 | 4 |
| 6. Teachers care about their students. | 1 | 2 | 3 | 4 |
| 7. Consequences of breaking rules are fair. | 1 | 2 | 3 | 4 |
| 8. In this school, students threaten and bully other students. | 1 | 2 | 3 | 4 |
| 9. Students know what they should do in school. | 1 | 2 | 3 | 4 |
| 10. Students are friendly towards most other students. | 1 | 2 | 3 | 4 |
| 11. In this school, bullying the weak has become a campus problem. | 1 | 2 | 3 | 4 |
| 12. Students are worried about being bullied by others at school. | 1 | 2 | 3 | 4 |
| 13. Students know what the rules are. | 1 | 2 | 3 | 4 |
| 14. Students care about each other. | 1 | 2 | 3 | 4 |
| 15. Teachers listen to students' doubts and difficulties. | 1 | 2 | 3 | 4 |
| 16. The school's code of conduct is fair. | 1 | 2 | 3 | 4 |
| 17. Students feel safe in this school. | 1 | 2 | 3 | 4 |
| 18. The school informed the students about how they should behave. | 1 | 2 | 3 | 4 |
| 19. Adults working in schools care about their students. | 1 | 2 | 3 | 4 |
| 20. Most of the students abide by the school rules. | 1 | 2 | 3 | 4 |
| 21. Students know they are safe in this school | 1 | 2 | 3 | 4 |
| 22. Most students hand in their homework. | 1 | 2 | 3 | 4 |
| 23. The classroom rules are fair. | 1 | 2 | 3 | 4 |
| 24. Most of the students study hard to get good grades. | 1 | 2 | 3 | 4 |
| 25. Students treat each other with respect. | 1 | 2 | 3 | 4 |
| 26. Students get along with each other. | 1 | 2 | 3 | 4 |
| 27. Students like their teachers. | 1 | 2 | 3 | 4 |
| 28. Teachers like their students. | 1 | 2 | 3 | 4 |

**(4) Depression**

| **Instructions:**  Please read each statement below carefully. Indicate how frequently you have experienced each of the following feelings or behaviors during the past week, including today. Circle the number that best corresponds to your response using the scale provided. There are no right or wrong answers. | **Rarely or none of the time（Less than 1 day）** | **Some or a little of the time（1-2 days）** | **Often（3-4 days）** | **Most or all of the time****（5-7 days）** |
| --- | --- | --- | --- | --- |
| 1. I was bothered by things that usually don’t bother me. | 1 | 2 | 3 | 4 |
| 2. I had trouble keeping my mind on what I was doing. | 1 | 2 | 3 | 4 |
| 3. I felt depressed. | 1 | 2 | 3 | 4 |
| 4. I felt that everything I did was an effort. | 1 | 2 | 3 | 4 |
| 5. I felt hopeful about the future. | 1 | 2 | 3 | 4 |
| 6. I felt fearful. | 1 | 2 | 3 | 4 |
| 7. My sleep was restless. | 1 | 2 | 3 | 4 |
| 8. I was happy. | 1 | 2 | 3 | 4 |
| 9. I felt lonely. | 1 | 2 | 3 | 4 |
| 10. I could not get “going.” | 1 | 2 | 3 | 4 |
